# Constructing a malaria-related health service readiness index and assessing its association with child malaria mortality: an analysis of the Burkina Faso 2014 SARA data

**DOI:** 10.1186/s12889-020-09994-7

**Published:** 2021-01-05

**Authors:** Ourohiré Millogo, Jean E. O. Doamba, Ali Sié, Jürg Utzinger, Penelope Vounatsou

**Affiliations:** 1 Swiss Tropical and Public Health Institute, Basel, Switzerland; 2 University of Basel, Basel, Switzerland; 3 Centre de Recherche en Santé de Nouna, Nouna, Burkina Faso

**Keywords:** Bayesian geostatistical models, Burkina Faso, Composite readiness index, Malaria, Service Availability and Readiness Assessment (SARA)

## Abstract

**Background:**

The Service Availability and Readiness Assessment surveys generate data on the readiness of health facility services. We constructed a readiness index related to malaria services and determined the association between health facility malaria readiness and malaria mortality in children under the age of 5 years in Burkina Faso.

**Methods:**

Data on inpatients visits and malaria-related deaths in under 5-year-old children were extracted from the national Health Management Information System in Burkina Faso. Bayesian geostatistical models with variable selection were fitted to malaria mortality data. The most important facility readiness indicators related to general and malaria-specific services were determined. Multiple correspondence analysis (MCA) was employed to construct a composite facility readiness score based on multiple factorial axes. The analysis was carried out separately for 112 medical centres and 546 peripheral health centres.

**Results:**

Malaria mortality rate in medical centres was 4.8 times higher than that of peripheral health centres (3.5% vs. 0.7%, *p* < 0.0001). Essential medicines was the domain with the lowest readiness (only 0.1% of medical centres and 0% of peripheral health centres had the whole set of tracer items of essential medicines). Basic equipment readiness was the highest. The composite readiness score explained 30 and 53% of the original set of items for medical centres and peripheral health centres, respectively. Mortality rate ratio (MRR) was by 59% (MRR = 0.41, 95% Bayesian credible interval: 0.19–0.91) lower in the high readiness group of peripheral health centres, compared to the low readiness group. Medical centres readiness was not related to malaria mortality. The geographical distribution of malaria mortality rate indicate that regions with health facilities with high readiness show lower mortality rates.

**Conclusion:**

Performant health services in Burkina Faso are associated with lower malaria mortality rates. Health system readiness should be strengthened in the regions of Sahel, Sud-Ouest and Boucle du Mouhoun. Emphasis should be placed on improving the management of essential medicines and to reducing delays of emergency transportation between the different levels of the health system.

**Supplementary Information:**

The online version contains supplementary material available at 10.1186/s12889-020-09994-7.

## Background

Over the past 20 years, considerable progress has been made in the fight against malaria. Indeed, there was an estimated reduction of 41% of clinical malaria incidence, and an estimated reduction in malaria mortality rate of 69% [1]. This success is mainly explained by the scaling up of cost-effective health interventions, such as insecticide-treated nets (ITNs), indoor residual spraying (IRS) and artemisinin-based combination therapy (ACT) [2]. Globally, 19 countries eliminated malaria and six of them have been certified malaria-free [1]. Notwithstanding, malaria remains a major public health issue in sub-Saharan Africa. Indeed, in 2017, 92% of the 219 million new cases of malaria and 93% of the 435,000 attributable deaths worldwide occurred in this part of the world. The disease burden is particularly high in children under the age of 5 years [1]. Burkina Faso accounts for 4 and 6% of the global clinical malaria incidence and malaria-related deaths, respectively. The Malaria Indicator Survey of 2014 estimated that the prevalence of malaria parasitaemia determined by rapid diagnostic tests (RDTs) was 61%, compared to 76% in 2010 [3].

The importance of health systems strengthening to reach health-related goals and targets is stressed since the early 2000s [4, 5]. Human resource shortages and inadequate training, poor supply chain management, inadequate infrastructure and equipment, and weak health information systems prevent the health facilities from responding adequately to populations needs [6–8]. Consequently, existing tools and strategies, designs and frameworks need to be improved in order to strengthen health systems [8–10]. In sub-Saharan Africa, only few counties regularly implement health systems assessment. In early 2010, the World Health Organization (WHO) developed the Service Availability and Readiness Assessment (SARA) survey to assess the readiness of health facilities to respond to community needs [11]. SARA surveys collect a set of binary tracer items on several domains related to the availability of basic equipment, basic amenities, essential medicines, diagnostic capacity and delivery of health interventions. The data cover readiness of health facilities to provide general services as well as services related to 20 health programmes, including malaria, HIV, tuberculosis, antenatal care, family planning and non-communicable diseases (NCDs).

Several authors have analysed the SARA survey tool and similar methodologies proposing statistical approaches to create a measure of health facility readiness and to relate readiness to health outcomes. Shawon and colleagues (2018), in their study following WHO guidelines, calculated separate readiness scores for each tracer item as the proportion of health facilities possessing the item [11, 12]. Domain-specific readiness scores for general (e.g. basic amenities, basic equipment, standard precautions for infection prevention, diagnostic capacity and essential medicines) and for malaria-specific services (e.g. staff and guidelines, diagnostics, medicines and commodities) were also calculated as the mean availability of the tracer items belonging to the domain. A similar approach has been adopted by Kanyangarara et al. (2018) to assess obstetric service readiness in 17 low- and middle-income countries (LMICs) [13]. Ali et al. (2018) obtained a general service score as the average of domain-specific scores to compare family planning service availability and readiness in 10 African countries [14]. This average composite measure takes into account the different aspects of health facility readiness. However, it assumes an equal contribution of the tracer items to the overall readiness. Boyer and colleagues (2015) applied principal component analysis (PCA) on the tracer items and defined a readiness index based on the first principal component. The index was utilized to assess the association between facility readiness with child survival, low birth weight, maternal and neonatal death in Ghana [15]. PCA has been applied to relate general service readiness and health financing factors in 10 countries in Africa and Asia [16], health facility readiness to child delivery services and service utilization in Haiti [17] or to assess facility readiness to maternal health services over time in Nigeria [18]. Of note, Ssempiira et al. (2019) criticized the use of PCA on binary items and derived a readiness index based on multiple correspondence analysis (MCA) [19]. To obtain a meaningful readiness score ensuring that the absence of any tracer item from a facility will contribute to a lower score than its presence, the authors proposed a composite measure based on more than one MCA axis.

SARA survey data from Burkina Faso have been used to assess readiness of surgical [20], obstetric [13] and family planning services [14]. However, no studies have been carried out to date to investigate the relationship between health service readiness and health outcomes in Burkina Faso. Hence, to fill this gap, we focused our research on malaria-related services and determined the extent to which malaria services readiness is effective and able to prevent malaria deaths in children under the age of 5 years. Our findings will help to optimize resources allocation and improve SARA survey analyses for Burkina Faso and other LMICs.

## Methods

### Study area and national health system

Malaria is endemic in Burkina Faso. It is the leading cause of health care consultation, hospitalisation and mortality in under 5-year-old children [21]. The health system of Burkina Faso is pyramidal and consists of three levels [22]. The peripheral level is formed by the health district and includes the “Centre de Santé et de Promotion Sociale” (CSPS), medical centres, isolate dispensaries, delivery centres and district hospitals. The latter serve as referral centres of the former health facilities. The second level is made of the regional hospitals, which are the reference structures for the district hospitals. The third level comprises the national and teaching hospitals and is the highest level of referral care providing specialized services. In 2016, there were approximately 1760 CSPS, 47 district hospitals, eight regional hospitals and five national and teaching hospitals.

### Data sources

#### The 2014 SARA survey

We analysed health facility data from the Burkina Faso SARA survey carried out in 2014 that included 786 health facilities grouped in three strata: (i) 19 teaching hospitals, private polyclinics and regional hospitals (stratum 1); (ii) 90 district hospitals and medical centres (stratum 2); and (iii) 671 CSPS, isolate dispensaries and delivery centres (stratum 3). Strata 1 and 2 correspond to a rather homogeneous group as they are staffed with physicians (in most cases), and hence, we combined them to increase the sample size and created two hierarchical levels of health facilities: medical centres (highest level) consisting of strata 1 and 2 and peripheral health centres (lowest level), including those of stratum 3. Of note, medical centres are usually staffed by physicians, while peripheral health centres are primarily managed by nurses.

The items in the SARA questionnaire are specific to the services provided by the health facilities and remain the same across health facility levels for a specific service. As facility levels differ in terms of the services and health programmes they offer, the items have different importance or weights depending on the facility level. For example, access to power grid is mostly found in medical centres as they are situated mainly in urban areas, while solar power is the main source of energy in rural areas. Medicines for chronic diseases or surgery, anesthesia and X-ray equipment are mainly part of the medical centres rather than peripheral health centres*.*

We defined as tracer items readiness indicator (i) for the general services and (ii) for the malaria-specific services, the proportion of health facilities having the tracer item available. The services were defined as binary variables taking the value “1” if the tracer item was available in the facility and “0” otherwise. Furthermore, we created domain readiness indicators for general (i.e. basic amenities, basic equipment, standard precautions for infection prevention, diagnostic capacity and essential medicines) and malaria services (i.e. staff and guidelines, diagnostics, medicines and commodities). Domain readiness indicators correspond to the proportion of health facilities having the whole set of tracer items belonging in the domain. We used “1” if all tracer items belonging to the domain where available at the health facility and “0” otherwise.

#### Health outcome: malaria-related mortality among under-5-year-old

Mortality data were extracted from the Health Management Information System (HMIS) for a full year (January–December 2014). Malaria mortality in children below the age of 5 years was defined as the number of malaria-related deaths among all in-patient visits to a health facility of that age group. The mortality outcome was linked to the SARA database according to the health facility.

### Statistical analysis

Bayesian negative binomial models were fitted on the number of malaria-related deaths at the health facility. We assumed that the number of malaria-related deaths at the health facility follows a negative binomial count distribution, and hence, Bayesian negative binomial models were fitted on the malaria deaths data. The total number of children below the age of 5 years visiting the facility (i.e. the denominator of the mortality rate outcome) was considered as an offset term in the model, that is the logarithmic transformation of it was introduced as a covariate with fixed regression coefficient equal to 1. The tracer items were included as covariates in the model. Bayesian variable selection was applied to determine the most important tracers associated with the malaria mortality rate. A separate analysis was carried out for each facility level, i.e. medical centres and peripheral health centres.

MCA was applied to the most important tracers, adhering to an approach put forth by Ssempiira et al. (2018) [19]. In short, let *K* be the set of selected tracers, *X*^*k*^, *k* = 1, …. *K* and ${X}_{0,i}^k$ and ${X}_{1,i}^k$ be two binary indicators corresponding to the presence or absence of the *X*^*k*^ from the facility *i*, respectively, that is, ${X}_{0,i}^k$ takes value 1 when the tracer *k* is absent (${X}_i^k=0$) and 0 otherwise. Likewise, ${X}_{1,i}^k$ takes value 1 when the tracer *k* is present in health facility *i* (i.e. ${X}_i^k=1$) and 0 otherwise.

The readiness score for health facility *i*, based on the *a*^*th*^ factorial axis is defined by ${F}_i^a=\frac{1}{K}\sum \limits_{k=1}^K\sum \limits_{j_k=0}^1{\mathsf{W}}_{j_k}^{a,k}{X}_{j_k,i}^k,$ where *j*_*k*_ indicates the value of *X*^*k*^ and the weights ${\mathsf{W}}_{j_k}^{a,k}$ are the columns standards coordinates on the *a*^*th*^ factorial axis corresponding to ${X}_{j_k,i}^k.$ Following the procedure of Asselin (2009), we define a composite readiness score as ${F}_i^a=\frac{1}{K}\sum \limits_{k=1}^K\ \sum \limits_{j_k\in \left\{0,1\right\}}^1\sum \limits_{a=1}^L\delta \left(k-a\right)\ {\mathsf{W}}_{j_k}^{a,k}{X}_{j_k,i}^k,$ where *L* is the number of factorial axes used in the composite score and *δ*(*k* − *a*) is the Dirac delta function, which takes the value 1 when the weights related to ${X}_{j_k,i}^k$ are selected from the factorial axis and 0 otherwise, that is, *δ*(*k* − *a*) = 1 if *k* = *a* and *δ*(*k* − *a*) = 0 if *k* ≠ *a*. The factorial axes that will represent the *X*^*k*^ tracer are identified based on a discrimination measure, which is calculated for each tracer and axis and measures the contribution of the tracer to the total variance explained by the axis. To improve interpretation of the score, we translated the weights so that the absence category *j*_*k*_ = 0 of the *X*^*k*^ tracer received a zero weight and the presence one *j*_*k*_ = 1 received a strictly positive weight indicating the gain in the readiness increase measured by the axis *a* when a facility *i* acquires the *k*^*th*^ tracer. Hence, the ${\mathsf{W}}_{j_k}^{a,k}$ in *F*_*i*_ is replaced by ${\mathsf{W}}_{j_k}^{+a,k}$, where ${\mathsf{W}}_0^{+a,k}=0$ and ${\mathsf{W}}_1^{+a,k}={\mathsf{W}}_1^{+a,k}-{\mathsf{W}}_1^{+a,k}$ [23]. The composite readiness score was converted into a readiness index with three categories by dividing the ordered distribution of the score values into three parts, each containing a third of the values.

Furthermore, we assessed the association between malaria mortality rate and the readiness index described above, using a geostatistical Bayesian negative binomial model. Locational random effects were included in the model to take into account spatial correlation. We assumed a Gaussian process with an exponential correlation function of the distance between health facilities. The analysis was adjusted for the type of health facility location (urban or rural) and of administrative status (publicor private). Further details of the statistical methods are provided in Additional file 1.

The descriptive analyses were carried out in STATA version 14 (StataCorp.; College Station, TX, USA) and Bayesian models were fitted in OpenBUGS version 3.2.3 (Imperial College and Medical Research Council; London, UK). Maps were produced in ArcGIS version 10.2.1 (Esri Inc.; Redlands, CA, USA).

## Results

### Health facility characteristics and malaria mortality

The SARA survey carried out in Burkina Faso in 2014 included 786 health facilities. Among these health facilities, 658 (83.7%) reported complete malaria mortality data, and hence, they were used for subsequent analyses. Seventeen percent of the facilities (*n* = 112) belonged to medical centres. Around 80% of medical centres are located in urban areas, while in peripheral health centres, more than 80% of the facilities are in rural zones (Table 1). Most of the facilities are managed by the government (77% of medical centres and 93% of peripheral health centres). The malaria mortality rate in medical centres is 4.8 times higher than that of peripheral health centres (3.5% vs 0.7%, *p* < 0.0001).

**Table 1:** Health facility characteristics and malaria mortality rates according to the SARA survey of 2014 in Burkina Faso

Characteristics	Medical centres (*n* = 112) n (%)	Peripheral health centres (*n* = 546) n (%)
Location		
Urban	90 (80.4)	83 (15.2)
Rural	22 (19.6)	463 (84.8)
Administrative management		
Public	86 (76.8)	510 (93.4)
Private	26 (23.2)	36 (6.6)
Regions		
Boucle du Mouhoun	9 (8.0)	65 (11.9)
Cascades	4 (3.6)	25 (4.6)
Centre	27 (24.1)	54 (9.9)
Centre-Est	10 (8.9)	38 (7.0)
Centre-Nord	6 (5.4)	41 (7.5)
Centre-Ouest	11 (9.8)	53 (9.7)
Centre-Sud	4 (3.6)	30 (5.6)
Est	9 (8.0)	40 (7.3)
Hauts Bassins	9 (8.0)	55 (10.1)
Nord	8 (7.1)	53 (9.7)
Plateau Central	4 (3.6)	38 (7.0)
Sahel	4 (3.6)	27 (5.0)
Sud-Ouest	7 (6.4)	27 (5.0)
Malaria		
Number of deaths (a)	1860	347
Number of consultations (b)	53,768	48,524
Mortality rate = a/b	3.5%	0.7%

### Domains and tracer items readiness’ indicators

Table 2 summarises the domains and tracer items readiness indicators of the general and malaria-specific services. Among the general service domains, basic equipment readiness was the most attainable domain (reached by 64.2 and 48.4% of medical centres and peripheral health centres, respectively). On the other hand, essential medicines was the domain with the lowest readiness (only 0.1% of medical centres and 0% of peripheral health centres had the whole set of essential medicines tracer items). Malaria services consisted of nine tracer items covering three domains. Apart of the diagnostic domain, which had one tracer, readiness of the staff and guidelines domain was higher in peripheral health centres compare to medical centres (57.7 and 45.5%, *p* = 0.027). Medicines and the commodities domain readiness was also higher in peripheral health centres but the difference to medical centres was borderline significant (31.5% vs 18.8%, *p* = 0.051).

**Table 2:** Frequency distribution of domains and tracer items readiness indicators as well as posterior inclusion probabilities of general and malaria-specific tracers estimated from the Bayesian variable selection. Tracers with inclusion probabilities higher than 50% were selected for the MCA

Domain/tracer items	Medical centres (*n* = 112)		Peripheral health centres (*n* = 546)	
	Availability (%)	Posterior inclusion probability^**2**^ (%)	Availability (%)	Posterior inclusion probability (%)
General service				
Basic amenities^1^	***39 (34.8)***		***6 (1.1)***	
Power (electric or solar device)	86 (76.8)	8.5	362 (66.3)	21.4
Improved water source inside or within the ground of the facility	110 (98.2)	−^**3**^	476 (87.2)	60.9
Room with auditory and visual privacy for patient consultations	81 (72.3)	100	284 (52.0)	39.2
Access to adequate sanitation facilities for clients	109 (97.3)	–	519 (95.1)	–
Communication equipment (phone or SW radio)	111 (99.1)	–	535 (98.0)	–
Facility has access to computer with E-mail/Internet access	56 (50.0)	6.9	10 (1.8)	–
Emergency transportation	106 (94.6)	61.7	515 (94.3)	88.0
Basic equipment	***72 (64.2)***		***264 (48.4)***	
Adult scale	108 (96.4)	–	527 (96.5)	–
Child scale	82 (73.2)	13.2	428 (78.4)	15.1
Thermometer	112 (100)	–	544 (99.6)	–
Stethoscope	112 (100)	–	540 (98.9)	–
Blood pressure apparatus	109 (97.3)	–	533 (97.6)	–
Light source	92 (82.1)	100	349 (63.9)	16.2
Standard precautions for infection prevention	***52 (46.4)***		***223 (40.8)***	
Safe final disposal of sharp materials	85 (75.9)	84.7	422 (77.3)	28.2
Safe final disposal of infectious wastes	82 (73.2)	62.9	336 (61.5)	18.2
Appropriate storage of sharp waste	110 (98.2)	–	535 (98.0)	–
Appropriate storage of infectious waste	103 (92.0)	85.3	494 (90.5)	50.8
Disinfectant	111 (99.1)	–	544 (99.6)	–
Single use (standard disposable or auto-disable syringes)	111 (99.1)	–	543 (99.5)	–
Soap and running water or alcohol based hand rub	105 (93.8)	33.6	518 (94.9)	99.2
Latex gloves	100 (89.9)	56.1	499 (91.4)	99.3
Guidelines for standard precautions	98 (87.5)	98.3	469 (85.9)	21.2
Diagnostic capacity	***37 (33.0)***		***3 (0.6)***	
Haemoglobin	72 (64.3)	100	9 (1.7)	–
Blood glucose	50 (44.6)	48.2	6 (1.1)	–
Malaria diagnostic capacity	101 (90.2)	17.5	467 (85.5)	21.3
Urine dipstick-protein	103 (92.0)	49.0	501 (91.8)	50.1
Urine dipstick-glucose	104 (92.9)	80.6	491 (89.9)	31.4
HIV diagnostic capacity	106 (94.6)	32.9	512 (93.8)	39.8
Urine test for pregnancy	96 (85.7)	26.0	412 (75.5)	42.3
Essential medicines	***2 (0.1)***		***0 (0)***	
Amoxicillin tablet	101 (90.2)	40.6	523 (95.8)	–
Ampicillin for inject	104 (92.9)	21.7	519 (95.1)	–
Gentamicin injectable	101 (90.2)	77.7	472 (86.5)	30.3
Oxytocin injectable	98 (87.5)	100	502 (91.9)	77.8
Amoxicillin dispersible	94 (83.9)	10.6	475 (87.0)	20.1
Oral rehydration solution (ORS)	95 (84.8)	16.8	476 (87.2)	20.3
Zinc	77 (68.8)	100	418 (76.6)	14.9
Aspirin	94 (83.9)	100	377 (69.1)	19.6
Magnesium sulfate	78 (69.6)	100	121 (22.2)	20.9
Amlodipine	25 (22.3)	100	12 (2.2)	–
Enalapril	20 (17.9)	26.1	6 (1.1)	–
Insulin injectable	8 (7.1)	35.9	5 (0.9)	–
Betablockers	20 (17.9)	100	8 (1.5)	–
Beclomethasone inhaler	14 (12.5)	100	9 (1.7)	–
Ceftriaxone injection	103 (92.0)	93.8	492 (90.1)	58.4
Thiazidic	25 (22.3)	14.2	41 (7.5)	50.6
Glibenclamide tablet	39 (34.8)	100	10 (1.8)	–
Metformin	41 (36.6)	22.9	9 (1.7)	–
Omeprazole	65 (58.0)	10.1	110 (20.2)	20.2
Salbutamol inhaler	86 (76.8)	63.3	288 (52.8)	24.9
Carbamazepine	28 (25.0)	69.9	0 (0.0)	–
Haloperidol	27 (24.1)	96.6	0 (0.0)	–
Simvastatin	4 (3.6)	–		
Fluoxetin	3 (2.7)	–		–
Malaria-specific service				
Staff and guidelines	***41 (45.5)***		***313 (57.7)***	
Guidelines for diagnosis and treatment of malaria	105 (93.8)	22.4	536 (98.2)	–
Guidelines for intermittent preventive treatment	75 (67.0)	13.0	481 (88.1)	31.1
Staff trained in malaria diagnosis and treatment	79 (70.5)	97.5	453 (83.0)	40.9
Staff trained in intermittent preventive treatment	74 (66.1)	100	370 (67.8)	58.9
Diagnostics	***101 (90.2)***		***467 (85.5)***	
Malaria diagnostic capacity (rapid diagnostic test/thin blood film)	101 (90.2)	17.5	467 (85.5)	21.3
Medicines and commodities	***21 (18.8)***		***172 (31.5)***	
First-line antimalarial in stock (artemether+lumefantrine, artesunate+amodiaqune)	99 (88.4)	58.8	526 (96.3)	–
Paracetamol cap/tab	104 (92.9)	100	418 (76.2)	34.6
Intermittent preventive treatment of malaria in pregnancy (IPTp) drug (sulfadoxine pyrimethamine)	62 (55.4)	28.4	356 (65.2)	17.1
ITNs	29 (25.9)	73.2	185 (33.9)	26.2

^1^Domain readiness indicators were defined as availability of all tracer items belonging to the domain

^2^Posterior inclusion probability: gives the probability of the tracer to be included in the final model and it is calculated by the proportion of all possible models in the variable selection procedure that include the specific tracer. For example, the posterior inclusion probability of 21.4 estimated for the power tracer indicates that this tracer was included in 21.4% of all possible models generated from all general services-related tracers

^**3**^Item not included in the variable selection procedure due to low relative frequency i.e. < 5%

Bayesian variable selection identified 29 tracers that are related to malaria deaths out of the 49 items across all domains of the general service offered by medical centres (Table 2). These are privacy room and emergency transportation (under basic amenities), light source (basic equipment), safe disposal of sharp materials, safe disposal and storage of infectious wastes, latex gloves and precaution guidelines (standard precautions for infection prevention), haemoglobin and glucose in urine (diagnostic), medicines for the management of NCDs (diabetes, cardiovascular and respiratory chronic diseases) and availability of two antibiotics (gentamycin and ceftriaxone) commonly used in medical centres (essential medicines). Five out of nine tracer items were selected in the malaria-specific service of medical centres (i.e. staff trained in malaria diagnostic and treatment, trained in intermittent preventive treatment of malaria, the first line of malaria treatment, paracetamol and ITNs).

For peripheral health centres, 29% (10/34) tracers were selected in the general service. These are similar to those in medical centres with the exception of the essential medicines, as most of them were not available in peripheral health centres. Regarding malaria-specific services offered by peripheral health centres, readiness to the first line of antimalarial drugs (96.3%) and to malaria diagnostics (85.5%) was similar as observed in medical centres.

### Health facility readiness index

MCA was applied on the tracers items selected from the variable selection procedure to obtain a readiness score. Fourteen and six factorial axes were sufficient to build the composite indices for medical centres and peripheral health centres, respectively. Standard coordinates of the selected tracers are provided in Table 3 (medical centres) and Table 4 (peripheral health centres).

**Table 3:** Standard coordinates of tracer items on the first 14 factorial axes (medical centres) derived from the SARA survey in 2014 in Burkina Faso.

Tracers	Category	Frequency, n(%)	Factorial axes^*^													
			1	2	3	4	5	6	7	8	9	10	11	12	13	14
Privacy room	No	31 (27.7)	**−0.281**^**a**^	**−1.196**	0.358	**−2.537**	1.257	**−1.370**	**−0.195**	0.746	0.171	***−5.265***	**−3.066**	1.560	0.252	1.213
	Yes	81 (72.3)	**0.107**	**0.458**	−0.137	**0.971**	−0.481	**0.524**	**0.075**	−0.285	− 0.065	***2.015***	**1.173**	−0.597	−0.097	− 0.464
Emergency transportation	No	6 (5.4)	0.063	**−3.332**	**−2.716**	4.195	0.012	**−7.946**	**4.844**	***−11.193***	**−0.109**	**− 1.787**	1.657	3.354	**−2.609**	2.616
	Yes	106 (94.6)	−0.004	**0.189**	**0.154**	−0.237	−0.001	**0.450**	**−0.274**	***0.634***	**0.006**	**0.101**	−0.094	−0.190	**0.148**	−0.148
Light power	No	20 (17.9)	**−0.925**	***−3.350***^***b***^	0.885	0.805	0.298	2.439	**−4.057**	**−1.751**	**−0.649**	**−3.378**	2.240	**−4.542**	**−0.070**	**− 0.001**
	Yes	92 (82.1)	**0.201**	***0.728***	−0.192	−0.175	− 0.065	−0.530	**0.882**	**0.381**	**0.141**	**0.734**	−0.487	**0.987**	**0.015**	**0.000**
Safe final disposal of sharps	No	27 (24.1)	1.254	**−0.656**	***−4.856***	**−2.973**	**− 0.352**	0.664	**− 0.421**	**− 0.995**	1.152	**− 0.588**	2.051	**− 0.445**	**− 0.888**	**− 0.100**
	Yes	85 (75.9)	−0.398	**0.208**	***1.542***	**0.944**	**0.112**	−0.211	**0.134**	**0.316**	−0.366	**0.187**	−0.652	**0.142**	**0.282**	**0.032**
Safe final disposal of infectious wastes	No	30 (26.8)	0.859	**−0.684**	***−4.958***	**−2.727**	0.017	0.163	**−1.408**	**− 0.591**	1.160	0.798	**−0.069**	0.185	**−0.102**	**− 0.391**
	Yes	82 (73.2)	−0.314	**0.250**	***1.814***	**0.998**	−0.006	−0.060	**0.515**	**0.216**	−0.424	−0.292	**0.025**	−0.068	**0.037**	**0.143**
Appropriate storage of infectious waste	No	9 (8.0)	1.198	**−2.643**	0.035	1.036	**−1.691**	***−9.057***	**− 1.236**	**− 0.570**	**− 1.376**	2.107	5.636	**−5.990**	3.825	3.190
	Yes	103 (92.0)	−0.105	**0.231**	− 0.003	− 0.091	**0.148**	***0.791***	**0.108**	**0.050**	**0.120**	−0.184	−0.492	**0.523**	−0.334	− 0.279
Latex gloves	No	12 (10.1)	**−0.252**	**−3.347**	0.867	0.983	**−4.095**	**−0.858**	***−6.782***	3.797	**−3.464**	0.525	2.358	0.705	**−3.537**	**−1.541**
	Yes	100 (89.9)	**0.030**	**0.402**	−0.104	− 0.118	**0.491**	**0.103**	***0.814***	−0.456	**0.416**	−0.063	−0.283	− 0.085	**0.424**	**0.185**
Guidelines for standard precautions	No	14 (22.5)	***−3.610***	0.980	**−1.949**	**−1.532**	**− 1.850**	**− 1.734**	4.077	1.023	**−2.052**	**− 3.909**	**− 0.936**	**− 3.326**	**− 1.546**	1.195
	Yes	98 (87.5)	***0.516***	−0.140	**0.278**	**0.219**	**0.264**	**0.248**	−0.582	− 0.146	**0.293**	**0.558**	**0.134**	**0.475**	**0.221**	−0.171
Haemoglobin test	No	40 (35.7)	**−1.086**	**−1.396**	**−0.563**	0.630	**−2.200**	0.331	0.568	**−0.251**	**− 2.686**	1.100	**− 0.855**	1.830	***−3.237***	0.473
	Yes	72 (64.3)	**0.603**	**0.775**	**0.313**	−0.350	**1.222**	−0.184	− 0.316	**0.139**	**1.492**	−0.611	**0.475**	−1.017	***1.798***	−0.263
Glucose dipstick	No	8 (7.1)	**−2.850**	0.482	**−4.354**	0.289	***−6.397***	2.569	3.601	4.457	**−1.015**	**−5.601**	0.354	**−5.051**	**−0.402**	**− 0.182**
	Yes	104 (92.9)	**0.219**	−0.037	**0.335**	−0.022	***0.492***	− 0.198	− 0.277	− 0.343	**0.078**	**0.431**	− 0.027	**0.389**	**0.031**	**0.014**
Amlopdipin	No	87 (77.7)	**−0.329**	**− 0.723**	0.354	**− 0.378**	**− 0.280**	**− 0.416**	**− 0.848**	0.125	1.106	0.339	**− 0.459**	0.036	0.424	0.184
	Yes	25 (22.3)	**1.144**	**2.515**	−1.231	**1.315**	**0.974**	**1.448**	**2.953**	−0.436	− 3.848	−1.180	**1.597**	−0.125	− 1.477	−0.640
Aspirin	No	18 (16.1)	***−3.484***	3.093	**−0.109**	**− 0.623**	1.267	**−1.229**	0.818	2.121	**−2.599**	1.476	0.217	**− 2.705**	1.985	1.667
	Yes	94 (83.9)	***0.667***	−0.592	**0.021**	**0.119**	−0.243	**0.235**	−0.157	− 0.406	**0.498**	− 0.283	− 0.042	**0.518**	− 0.380	− 0.319
Beclomethasone inhaler	No	98 (87.5)	**−0.205**	**−0.683**	0.214	**−0.707**	**− 0.065**	**− 0.013**	0.293	**− 0.313**	**− 0.362**	0.313	0.484	0.025	0.342	**−0.079**
	Yes	14 (12.5)	**1.435**	**4.781**	−1.495	**4.948**	**0.452**	**0.094**	−2.052	**2.190**	**2.536**	− 2.189	−3.386	−0.177	− 2.394	**0.555**
Beta-blockers	No	92 (82.1)	**−0.433**	**− 0.793**	0.614	**−0.473**	**− 0.185**	0.294	0.561	**− 0.134**	0.132	**−0.074**	0.098	0.032	0.439	0.587
	Yes	20 (17.9)	**1.990**	**3.648**	−2.824	**2.176**	**0.849**	−1.355	−2.581	**0.616**	−0.609	**0.339**	− 0.450	− 0.145	− 2.021	− 2.701
Ceftriaxone	No	9 (8.0)	***−6.392***	1.090	0.991	**−0.869**	3.199	**−1.868**	**−2.313**	**−2.918**	2.354	**−0.844**	3.647	1.036	**−6.562**	**−1.918**
	Yes	103 (92.0)	***0.558***	−0.095	− 0.087	**0.076**	− 0.280	**0.163**	**0.202**	**0.255**	−0.206	**0.074**	−0.319	− 0.090	**0.573**	**0.168**
Gentamicin	No	11 (9.8)	***−4.331***	**−1.279**	**−2.234**	4.478	2.838	2.256	**−0.629**	**− 0.754**	1.864	1.838	**−1.003**	**−2.284**	**−0.105**	6.498
	Yes	101 (90.2)	***0.472***	**0.139**	**0.243**	−0.488	− 0.309	− 0.246	**0.069**	**0.082**	− 0.203	− 0.200	**0.109**	**0.249**	**0.011**	−0.708
Glibenclamide	No	73 (65.2)	**−0.724**	**−0.211**	**− 0.581**	0.607	0.655	0.053	**−0.176**	**−0.305**	**−1.378**	**− 0.786**	0.346	0.540	1.845	***−1.922***
	Yes	39 (34.8)	**1.356**	**0.395**	**1.088**	−1.137	− 1.225	− 0.099	**0.329**	**0.571**	**2.580**	**1.471**	−0.648	−1.011	−3.453	***3.598***
Insulin injectable	No	104 (92.9)	**−0.123**	***−0.424***	0.222	**−0.186**	**− 0.106**	**−0.147**	**0.493**	0.038	0.378	0.233	**−0.339**	**− 0.364**	**− 0.207**	**−0.641**
	Yes	8 (7.1)	**1.596**	***5.512***	−2.880	**2.420**	**1.375**	**1.909**	**−6.404**	−0.489	−4.911	−3.035	**4.404**	**4.737**	**2.688**	**8.330**
Magnesium	No	34 (30.4)	***−2.083***	**− 2.028**	**−0.340**	1.431	0.424	0.597	**−1.968**	0.063	0.339	**−0.313**	**−2.323**	0.905	0.300	**−0.988**
	Yes	78 (69.6)	***0.908***	**0.884**	**0.148**	−0.624	− 0.185	− 0.260	**0.858**	−0.028	− 0.148	**0.136**	**1.013**	−0.395	− 0.131	**0.431**
Oxytocin	No	14 (12.5)	***−3.089***	**−0.951**	**−3.370**	5.102	**−0.779**	2.837	**1.260**	**−2.386**	2.970	1.110	**−1.926**	**− 2.982**	0.951	**−0.272**
	Yes	98 (87.5)	***0.441***	**0.136**	**0.481**	−0.729	**0.111**	−0.405	**− 0.180**	**0.341**	− 0.424	− 0.159	**0.275**	**0.426**	− 0.136	**0.039**
Salbutamol	No	26 (23.2)	***−3.000***	**− 0.517**	**−1.091**	**−1.330**	**−0.915**	**− 0.662**	**− 0.223**	1.480	**−2.038**	1.850	**− 0.362**	1.727	0.879	1.003
	Yes	86 (76.8)	***0.907***	**0.156**	**0.330**	**0.402**	**0.277**	**0.200**	**0.067**	−0.448	**0.616**	− 0.559	**0.109**	− 0.522	− 0.266	− 0.303
Zinc	No	35 (31.3)	***−2.157***	0.502	**−0.495**	**−0.975**	0.011	2.273	**0.503**	**−0.816**	1.147	0.610	1.572	2.790	1.487	0.844
	Yes	77 (68.8)	***0.980***	−0.228	**0.225**	**0.443**	−0.005	−1.033	**− 0.229**	**0.371**	−0.522	− 0.277	− 0.715	− 1.268	− 0.676	− 0.383
ITNs	No	83 (74.1)	**−0.220**	0.045	**−0.747**	**− 0.247**	0.762	**−1.081**	**−0.434**	**− 0.005**	**− 0.520**	0.795	***− 1.332***	**− 0.377**	0.161	**− 0.081**
	Yes	29 (25.9)	**0.628**	−0.128	**2.139**	**0.708**	−2.180	**3.093**	**1.242**	**0.015**	**1.487**	−2.276	***3.811***	**1.079**	−0.461	**0.231**
Staff trained in malaria diagnosis and treatment	No	33 (29.5)	***−1.091***	1.604	**−0.341**	**− 0.226**	***−3.506***	**− 0.728**	**− 1.149**	1.928	2.217	**− 0.102**	0.246	0.246	1.343	**−0.185**
	Yes	79 (70 5)	***0.456***	−0.670	**0.143**	**0.094**	***1.465***	**0.304**	**0.480**	− 0.806	−0.926	**0.043**	− 0.103	− 0.103	− 0.561	**0.077**
Staff trained in intermittent preventive treatment in pregnancy (IPTp)	No	38 (33.9)	**−0.158**	**−0.459**	**− 0.829**	2.575	**−1.426**	***−2.856***	**0.619**	0.713	1.757	**−1.492**	1.060	2.415	0.922	0.162
	Yes	74 (66.1)	**0.081**	**0.236**	**0.426**	−1.322	**0.732**	***1.467***	**−0.318**	−0.366	− 0.902	**0.766**	− 0.544	−1.240	− 0.474	− 0.083
First line treatment of malaria	No	13 (11.6)	***−4.606***	2.906	0.766	**−1.353**	0.142	**−1.435**	**0.786**	0.550	2.369	1.904	2.504	0.379	**−0.856**	**−4.781**
	Yes	99 (88.4)	***0.605***	−0.382	− 0.101	**0.178**	− 0.019	**0.188**	**− 0.103**	− 0.072	−0.311	− 0.250	−0.329	− 0.050	**0.112**	**0.628**
IPTp drug	No	50 (44.6)	***−5.589***	3.721	1.822	**−2.447**	5.322	**−2.872**	**− 2.985**	0.352	1.426	**−2.683**	3.527	**−0.455**	**−4.909**	2.079
	Yes	62 (55.4)	***0.430***	−0.286	− 0.140	**0.188**	− 0.409	**0.221**	**0.230**	−0.027	− 0.110	**0.206**	−0.271	**0.035**	**0.378**	−0.160
Carbamazepine	No	84 (75.0)	**−0.144**	0.764	0.367	**−0.450**	***−1.050***	0.110	**−0.594**	**−1.146**	**−0.364**	0.136	**−0.686**	0.030	0.566	0.637
	Yes	28 (25.0)	**0.432**	−2.292	−1.101	**1.349**	***3.149***	−0.331	**1.782**	**3.438**	**1.093**	−0.408	**2.057**	−0.091	−1.698	−1.911
Haloperidol	No	85 (75.9)	**−0.183**	0.825	0.330	**−0.199**	**− 0.894**	**− 0.172**	**− 0.410**	**−1.475**	**− 0.025**	**−0.361**	**− 0.402**	**− 0.354**	**− 0.078**	**− 0.660**
	Yes	27 (24.1)	**0.577**	−2.599	−1.039	**0.627**	**2.815**	**0.541**	**1.292**	**4.644**	**0.079**	**1.136**	**1.266**	**1.116**	**0.244**	**2.078**
**Inertia explained by the factorial axis (%)**			**14.5**	**8.9**	**6.7**	**6.1**	**5.9**	**5.3**	**4.7**	**4.4**	**4.0**	**3.7**	**3.6**	**3.4**	**3.0**	**2.9**

^*^First 14 factorial axes to build the composite readiness score as there is no information gain beyond axis 14

^a^Four tracers consistent with the FAOC-G in negative direction (not bold) and 25 consistent in positive direction (bold)

^b^Highlighted in bold and italic are the weights of tracers from factorial axes selected to build the composite readiness score

**Table 4:** Standard coordinates of tracer items on the first six factorial axes (peripheral health centres)

Tracers	Category	Frequency	Factorial axes^*^					
			1	2	3	4	5	6
Improved water source	No		0.457^a^	0.048	**−5.424**	0.301	***−5.699***	0.095
	Yes	476 (87.2)	−0.067	−0.007	**0.798**	−0.044	***0.838***	− 0.014
Emergency transportation	No		***−5.770***^***b***^	**−0.594**	**−1.284**	**−0.830**	**−1.483**	3.543
	Yes	515 (94.3)	***0.347***	**0.036**	**0.077**	**0.050**	**0.089**	−0.213
Soap or running water	No		**−1.239**	**−0.895**	8.502	**−5.784**	***−7.725***	**−0.620**
	Yes	518 (94.9)	**0.067**	**0.048**	−0.460	**0.313**	***0.418***	**0.034**
Storage infectious waste	No		0.602	***−6.612***	**− 0.633**	1.732	**−0.529**	0.274
	Yes	494 (90.5)	−0.063	***0.696***	**0.067**	−0.182	**0.056**	−0.029
Latex gloves	No		0.418	***−7.016***	1.337	1.780	**−0.218**	0.025
	Yes	499 (91.4)	−0.039	***0.661***	−0.126	− 0.168	**0.021**	− 0.002
Urine dipstick	No		***−4.999***	0.574	**−0.718**	1.596	**−1.019**	2.063
	yesYes	501 (91.8)	***0.449***	−0.052	**0.065**	−0.143	**0.092**	−0.185
Ceftriaxone	No		***−3.772***	**−1.310**	**− 1.489**	**− 1.567**	3.602	1.638
	Yes	492 (90.1)	***0.414***	**0.144**	**0.163**	**0.172**	−0.395	− 0.180
Oxytocin	No		***−5.750***	**−0.119**	1.406	0.905	**−0.920**	0.604
	Yes	502 (91.9)	***0.504***	**0.010**	−0.123	− 0.079	**0.081**	− 0.053
Thiazidic	No		0.048	**−0.190**	**−0.263**	***− 0.751***	0.136	0.041
	Yes	41 (7.5)	−0.586	**2.338**	**3.235**	***9.250***	−1.672	−0.510
IPTg training	No		**− 1.518**	**−0.266**	**− 0.552**	**− 0.122**	0.163	***−4.223***
	Yes	370 (67.8)	**0.722**	**0.127**	**0.263**	**0.058**	−0.077	***2.009***
**Inertia explained by the factorial axis (%)**			**20.0**	**14.2**	**10.5**	**10.3**	**9.9**	**8.8**

^*^First 6 factorial axes to build the composite readiness score as there is no information gain beyond axis 6

^a^Four tracers consistent with the FAOC-G in negative direction (not bold) and 6 consistent in positive direction (bold)

^b^Highlighted in bold and italic are the weights of tracers from factorial axes selected to build the composite readiness score

For medical centres, the factorial axis 1 accounted for 10 tracer items, followed by axis 2 with five tracer items. The most weighted rescaled tracer items were the emergency transportation and appropriate storage of infectious waste picked from factorial axes eight and six, respectively. On the first factorial axis, a subset of four tracers met the Global First Axis Ordering Consistency (FAOC-G) requirement in the positive direction, while a second subset of 25 tracer items met this condition in the negative direction (i.e. the score monotonically increases/decreases for all tracer items) [23]. Hence, there are two subsets of tracer items that are inconsistent and one subset should have been discarded, leading to a loss of information if we had constructed the score using the first factorial axis. With regard to peripheral health centres, four tracer items showed a high discrimination measure on factorial axis 1. The highest weighted tracers are “thiazidic” and “running water source or soap” from axes 4 and 5, respectively. The discrimination measures of the tracers and the rescaled weights are given in Tables 2.1 and 2.2 (in Additional file 2) for medical centres and peripheral health centres, respectively.

**Fig. 1:**
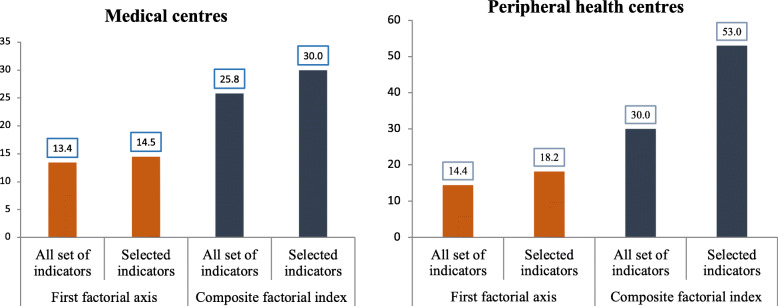
Proportion of variance explained by the first factorial axis (red) and the composite readiness score (blue) based on the whole set and the subset of tracers identified by the Bayesian geostatistical negative binomial models.

Figure 1 shows the proportion of variation in the tracers explained by the first factorial axis and the composite readiness score based on (i) the whole set of tracers and (ii) the subset of tracers identified by the Bayesian variable selection. The results show that the composite score explains more than twice the variance explained by the first factorial axis (medical centres: 30% vs. 15%; peripheral health centres 53% vs. 18%). Furthermore, the composite score based on the subset of tracers explained more variation than the composite score based on the whole set (medical centres: 30% vs. 26%; peripheral health centres: 53% vs. 30%).

### Association between health facility readiness and malaria mortality

The composite readiness score was converted into a categorical index with three categories defined by the tertiles of its distribution. Results of the Bayesian geostatistical negative binomial model fitted on malaria mortality indicated that medical centres with the highest and moderate readiness experienced a lower mortality rate by 19 and 6%, respectively, compared to the facilities with the lowest readiness (Table 5). However, this difference lacked statistical significance. The type of management and the location of health facilities do not influence malaria mortality.

**Table 5:** Posterior estimates (median and 95% BCI) of the association between health facility readiness and malaria mortality obtained from a Bayesian geostatistical negative binomial model

	Medical centres	Peripheral health centres
Readiness index	MRR^a^ (95% BCI)	MRR (95% BCI)
Low	1.00	1.00
Middle	0.94 (0.76–1.25)	0.74 (0.54–1.00)
High	0.81 (0.74–2.51)	0.41 (0.19–0.91)*
Location		
Rural	1.00	1.00
Urban	0.97 (0.48–1.77)	0.49 (0.31–0.78)*
Administrative status		
Private	1.00	1.00
Public	1.12 (0.51–2.17)	0.69 (0.46–1.01)
Spatial parameters		
Spatial variance	0.26 (0.14–0.53)	0.46 (0.29–0.67)
Spatial range (km)	43.3 (13.6–89.9)	26.32 (6.39–83.1)

^*a*^*MRR* Mortality rate ratio

*: Statistically important association

Peripheral health centres at the highest readiness category had a mortality rate ratio (MRR) of 0.41 (95% Bayesian credible interval (BCI): 0.19–0.91) compared to those with the lowest readiness. Furthermore, urban health facilities were associated with a statistically important reduction of malaria mortality compared to those in rural areas (MRR: 0.49, 95% BCI: 0.31–0.78). The median spatial range distance (distance over which the spatial correlation is no more important) was higher in medical centres compared to peripheral health centres.

**Fig. 2:**
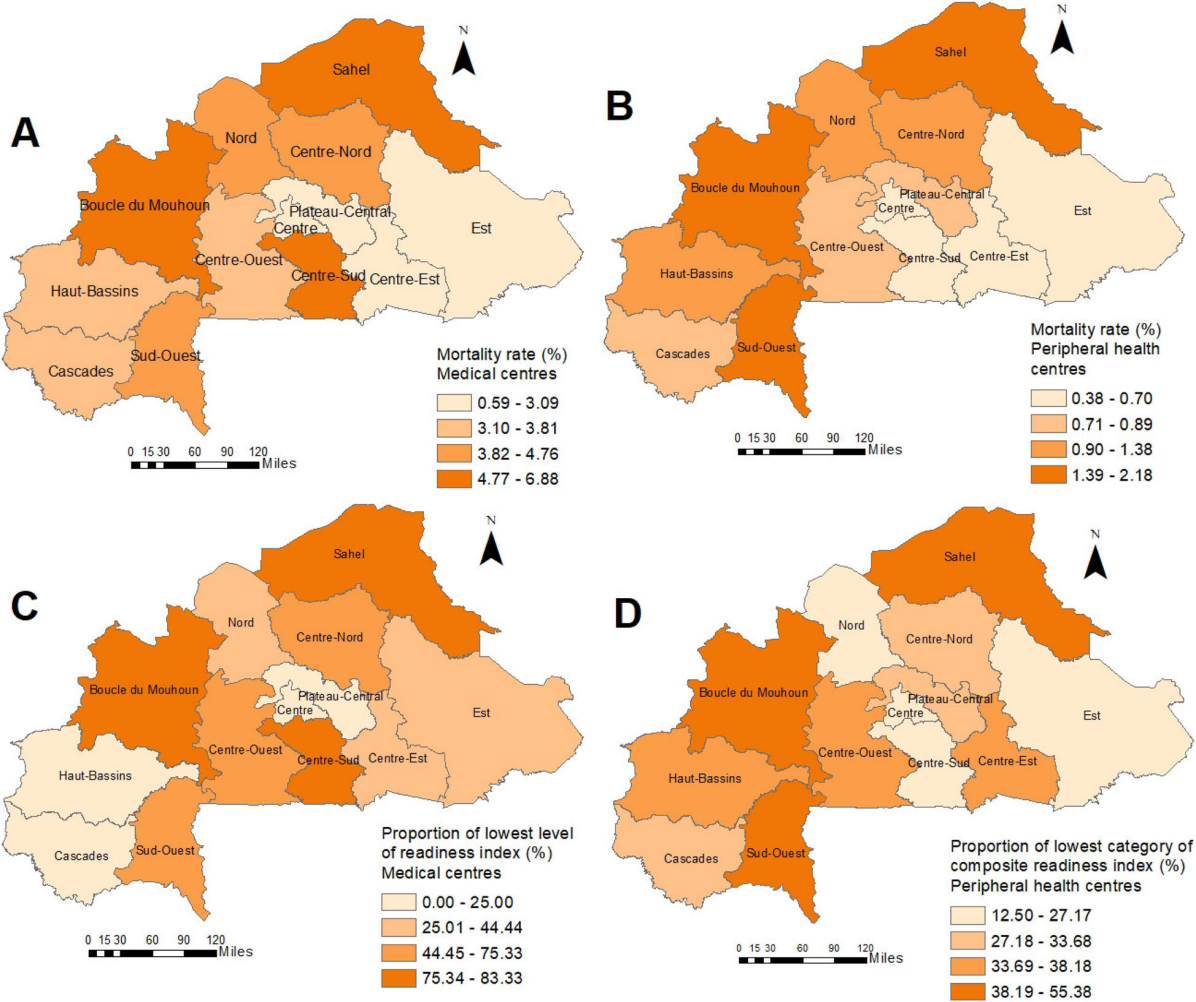
Spatial distribution of malaria-related mortality rate among children under the age of 5 years in Burkina Faso for medical centres (**a**) and peripheral health centres (**b**) and the proportion of health facilities medical centres (**c**) and peripheral health centres (**d**) in the lowest category of the corresponding composite readiness index

The geographical distribution of malaria mortality rate showed a similar pattern with that of the proportion of health facilities with lowest readiness (Fig. 2), indicating that regions with high malaria mortality rate have high proportion of facilities with low readiness and vice versa. In particular, the region of Centre (first region in terms of health infrastructure and population) showed for both health facility levels low malaria mortality rates, while Sud-Ouest, Sahel and Boucle du Mouhoun were those among the highest mortality and highest proportion of low performing facilities.

## Discussion

### Malaria services readiness and malaria-related mortality

The aim of our study was to estimate the extent to which malaria services readiness in Burkina Faso was associated with malaria mortality. Service delivery is an essential building block of the WHO health systems framework [8]. Our research indicated that the higher the readiness index, the lower the mortality in peripheral health centres. Hence, the index is sensitive enough to identify some of the barriers in the quality of the management of malaria cases. Information from Malaria Indicator Surveys and of the HMIS can be included as additional components of this index to look into other aspects of case management, such as delays of seeking care, the severity of cases consulting or the quality of care provided. Our results corroborate with previous investigations done in Bangladesh, Ghana, Haiti, Mozambique, Nigeriaand Tanzania that also used SARA or similar survey data and revealed a positive effect of readiness on health outcomes [15–18, 24, 25].

The lack of a statistically important association between facility readiness and malaria mortality in medical centres might be explained by the severity of malaria cases seeking treatment in medical centres. Indeed, peripheral health centres refer complicated cases to medical centres. Hence, although the latter are better equipped and staffed, the mortality rate is partially influenced by the seriousness of their cases. On the other hand, the reduced mortality rate in peripheral health centres with highest readiness was certainly related to prompt diagnosis and adequate treatment, since peripheral health centres receive patients at an early stage of the disease. This is consistent with the important association of the emergency transportation tracer with malaria mortality. In medical centres, emergency transportation obtained the highest weight. Reducing the delay of reference from peripheral health centres to medical centres will reduce the probability of deaths due to a severe malaria [26–29]. In addition, training health workers of peripheral health centres would allow for early reference decisions. At community level, populations must be encouraged to consult very early. In peripheral health centres, we noticed that medicines for NCDs management had low availability, although one drug devoted to chronic diseases had the highest weight. The low availability could be explained by an insufficiency in the supply of this type of drug and thus a low quality of the management of chronic diseases. On the contrary, its presence may mean competent health workers in the provision of drugs and thus a better quality of care and therefore to the management of malaria cases as well.

### Tracer items and domains readiness

Results of the individual tracers and domain readiness indicators are consistent with the role assigned to each level. Peripheral health centres are the first contact with any health issues and thus they provide the so called “minimum package” of health care and services, while medical centres provide the “complementary package”. Basic equipment was the most available domain for both levels of health care and for general services. The most widely available items within this domain were thermometer, stethoscope, adult scale and blood pressure apparatus, which represent minimum essential equipment to manage patients. However, their availability was almost 50% in peripheral health centres meaning that the quality of health care is not guaranteed in about half of the peripheral health centres, suggesting lack of financial resources and of management of supplies in peripheral health centres.

The weakest domain for both levels for general services was the essential medicine with an availability of less than 1%. Two types of medicines appeared in this domain; medicines for infectious diseases (availability > 80%) and medicines for chronic diseases (availability < 10%). The situation depicts the epidemiological profile of Burkina Faso, where infectious diseases are still predominant, but also indicates that services towards chronic diseases and NCDs in 2014 were inadequate, particularly in view of NCDs rapidly gaining importance in LMICs [21, 30–32]. This also indicates the weakness in the drug supply circuit of health facilities from the expression of adequate needs, to the availability of drugs at the point of purchase [33, 34].

The diagnostic capacity domain was very weak in peripheral health centres (0.6%) compared to medical centres (33%) even though in peripheral health centres, large number of biological diagnostic tests do not need sophisticated equipment. Peripheral health centres generally refer patients who need further biological testing. Nevertheless, the level of availability of malarial diagnosis capacities was > 80% appreciable in both levels and reflects the high workload relative to malaria in consultations [22].

The basic amenities domain is related to the health infrastructure investment and depends heavily on the financial support of the government. At the time of the SARA survey in 2014, only 1.9% of peripheral health centres had a computer. Hence, computers were the exception rather than the norm in peripheral health centres.

Regarding malaria-specific services, the average availability of “staff and guidelines” and the “medicine and commodity” domains was higher in peripheral health centres than medical centres. More than 80% of them had their staff trained and knew the guidelines for malaria management. In addition, more than 95% in these facilities possessed first-line treatment for malaria. Malaria is the most important cause of morbidity and mortality in under 5-year-old children, which explains that substantial efforts are being made to train peripheral health facility workers, render medicines and other medical supplies available for malaria case management at all levels of the health system. In recent years, there has been a shift from first-line medicines to ACTs, introduction of RDTs, and ITN campaigns [35, 36]. However, the availability of ITNs in health facilities had reduced the availability of malaria readiness in general because it is mostly during mass campaign that ITNs are distributed to pregnant women.

### Variables selection

The variable selection highlighted facts that are consistent with the health system in Burkina Faso. In both health facility levels and for general service readiness, “emergency transportation” was selected. In general, emergency transportation (ambulances) which reduces the delay to reach a health centre is available in medical centres. Peripheral health centres use mainly motorcycles for transportation. The malaria management policy in Burkina Faso requests that cases are confirmed before treatment; yet, there is still considerable empiric treatment [21]. Without a diagnostic test, malaria might be confused with other infectious diseases, which has ramifications on disease management, including treatment [37, 38]. This may explain the heavy prescription not only of antimalarials but also antibiotics, such as “gentamicin”and “ceftriaxone”.

### Geographical distribution of readiness and mortality rate

The geographical distribution of the under-5 malaria-related mortality corresponds almost to the HMIS statistics in 2014 suggesting that the regions of the Boucle du Mouhoun, Sahel and Sud-Ouest had the highest mortality rates and that malaria was the leading cause of deaths in this age group at that time. Regions with low mortality rates are concentrated in the central and eastern parts of the country for both levels. Apart from the fact that there is a greater concentration of health workers around the central region, there is no evidence to explain this distribution of mortality [21]. Similarly, to the mortality rate, the geographical distribution of the readiness index is heterogeneous for both levels. Nevertheless, the regions of Centre and Hauts Bassins are the best equipped and have the highest numbers of health facilities. They gather more than half of health human resources in Burkina Faso and possess most performant medical centres.

### Strengths and limitations

Our findings clearly favoured the construction of a composite readiness indicator rather than one derived from the first factorial axis. Indeed, the proportion of variance explained has more than doubled in both health facility levels compared to the first component. The composite index takes also into account the multifactorial and multidimensionality of the readiness allowing capturing tracers items that are represented better by high order axes. The variable selection identifies the subset of the most important tracers that are related to malaria mortality producing a score which explains even more variation in the tracers and it is directly related to a specific health outcome and thus, can led comprehensive policy decisions to strengthen the specific health services and care. The methodology can be applied on SARA or SARA-like survey in other countries.

However, SARA survey assess availability of items the day of the survey and thus do not take into account the variability over time of the items and 1 day may not be sufficient to get the mean availability of an item in a health facility longitudinally. The SARA proposed methodology weights all tracer items equally in the construction of readiness index; however, our proposed approach addresses this limitation. Unfortunately, mortality data in the HMIS were not available for several health facilities; therefore, we could not include data from those facilities in the analysis. Our results reflect the readiness of malaria services in Burkina Faso in 2014. The country has performed two more surveys in 2016 and 2018. Our methodology can be easily extended to construct a temporally varying readiness index and therefore assess potential improvements in the health facility malaria service provision.

## Conclusion

Our results indicate that investing in health services is an effective means for reducing the burden of malaria in Burkina Faso. The broad implication is that resources and efforts must be maintained and strengthened, particularly at medical centres where mortality rate is high and at weak peripheral health centres. The emergency transportation mechanisms between the different levels of the health system need to be further enhanced. The composite readiness score created by exploiting more than one MCA factorial axis produces a more informative and consistent health facility readiness measure that captures all aspects of readiness unlike the index based on only the first axis.

## Supplementary Information

**Additional file 1.** Supplementary information

**Additional file 2.** Supplementary Tables

## Data Availability

The SARA database and the HMIS database are accessible via request to the Department of Statistics of the Ministry of Health of Burkina Faso (zongoaugustin@yahoo.fr).

## Abbreviations

ACT: Artemisinin-based combination therapy
BCI: Bayesian credible interval
CSPS: Centre de Santé et de Promotion Sociale
FAOC-G: Global first axis ordering consistency
HMIS: Health management and information system
IPT: Intermittent preventive treatment of malaria
IRS: Indoor residual spraying
ITN: Insecticide-treated net
LMICs: Low- and middle-income countries
MCA: Multiple correspondence analysis
MRR: Mortality rate ratio
NCDs: Non-communicable diseases
ORS: Oral rehydration solution
PCA: Principal component analysis
RDT: Rapid diagnostic test
SARA: Service Availability and Readiness Assessment
WHO: World health Organization
