# Supplementary material for: Constructing a malaria-related health service readiness index and assessing its association with child malaria mortality: an analysis of the Burkina Faso 2014 SARA data
Source: BMC Public Health. 2021 Jan 5;21:20. doi: 10.1186/s12889-020-09994-7 (PMC7784320; doi:10.1186/s12889-020-09994-7)
Supplement: Supplementary file 1 — Additional file 1. Supplementary information [file 12889_2020_9994_MOESM1_ESM.docx]

**1.1: Model formulation**

A geostatistical negative binomial model [1] was fitted to assess the effect of the facility readiness index on malaria mortality adjusted for facility characteristics (type of health facility location and of administrative status). Let $Y_{i}$ be the number of malaria related deaths reported by health facility $i$ during January – December 2014. $Y_{i}$ is assumed to follow a negative binomial distribution, $Y_{i}\sim NB (p_{i}, r)$ where $p_{i}=\frac{r}{r +\mu_{i}}$and $r$is the dispersion parameter of the distribution. We relate the predictors to the mean count $\mu_{i}$ of the malaria mortality outcome reported at facility $i$ via the log-linear regression equation, $log\left( \mu_{i} \right)=log(N_{i})+\boldsymbol{\beta}^{T}\boldsymbol{X}+\omega_{i}$ +$\varphi_{i}$where $N_{i}$ is the offset which was considered to be the total number of children hospitalized due to malaria. $\boldsymbol{X}$ are the predictors, that is, the facility readiness index and facility characteristics, and $\boldsymbol{\beta}$ is the vector of regression coefficients. $\omega_{i}$ are facility location random effects added in the model to account for spatial dependence in the malaria mortality. We assumed a Gaussian process on $\boldsymbol{\omega}={(\omega_{1},\omega_{2},\ldots,\omega_{k})}^{T},$that is, $\boldsymbol{\omega}\sim N(0,\sigma^{2}R)$ where *R* is a correlation matrix, defined by an exponential parametric function of the distance $d_{ij}$ between the locations of facilities *i* and $j$ i.e. $R_{ij}=exp(-d_{ij}\rho)$. The parameter $\sigma^{2}$ measures the spatial variation and $\rho$ is a smoothing parameter that controls the rate of correlation decay with increasing distance. The range parameter, $\frac{3}{\rho}$ estimates the minimum distance beyond which spatial correlation is negligible. Non-spatial variation is estimated by the location random effects$\varphi_{i}$, which is assumed to be independent and normally distributed with mean 0 and variance $\sigma_{\varphi}^{2}$ , that is, $\varphi_{i}\sim N(0,\sigma_{\varphi}^{2})$. Model fit and parameter estimation was performed using Bayesian formulation and Markov Chain Monte Carlo (MCMC) estimation. Model specification was completed by assigning prior distributions to model parameters. An inverse-gamma hyperprior was assigned for the variance $\sigma_{\varphi}^{2}$, a gamma distribution for the spatial smoothing parameter, and non-informative Gaussian distributions for the regression coefficients with mean 0 and variance 100. Model parameters were estimated using MCMC simulation, running a two-chain algorithm with a burn-in of 10,000 iterations followed by 200,000 iterations. Convergence was formally assessed by the Gelman and Rubin diagnostic [2], implemented in CODA.

**1.2: Multiple correspondence analysis**

Let $K$ denote the number of binary tracer items, N be the number of health facilities and $\boldsymbol{X}_{Nx(2*K)}$ denote the indicator matrix in which the facilities are displayed as rows and each tracer is represented by the inclusion of two columns $\boldsymbol{I}_{j_{k}}^{k}$, one per category of the tracer $k=1,\ldots,K,$ corresponding to its presence $(j_{k}=1)$ or absence $(j_{k}=0)$ from the facility. Let $\boldsymbol{P}$ be the matrix $\boldsymbol{P}=\frac{1}{N*K}\boldsymbol{X}$**,** $r$ and $c$ the vectors of the row and column totals of $\boldsymbol{P}$, respectively, and $\boldsymbol{S}$ the matrix $\boldsymbol{S=}\boldsymbol{D}_{r}^{\boldsymbol{-}\frac{\boldsymbol{1}}{\boldsymbol{2}}}\boldsymbol{(P-r}\boldsymbol{c}^{T}\boldsymbol{)}\boldsymbol{D}_{r}^{\boldsymbol{-}\frac{\boldsymbol{1}}{\boldsymbol{2}}}$ where $\boldsymbol{D}_{r}=diag\{\boldsymbol{r}\}$ and $\boldsymbol{D}_{c}=diag\{c\}$. A readiness score $F_{i}^{a}$ corresponding to health facility $i$ and based on the $a^{th}$ factorial axis of MCA is defined by $F_{i}^{a}=\frac{1}{K}\sum_{k=1}^{K} \sum_{j_{k}\in\{0,1\}} W_{j_{k}}^{a,k}X_{j_{k},i}^{k}$ where the weights $W_{j_{k}}^{a,k}$ are the corresponding column standard coordinates of the $a^{th}$ factorial axis, that is, they are elements of the $a^{th}$ column of the matrix $\boldsymbol{D}_{c}^{\boldsymbol{-}\frac{\boldsymbol{1}}{\boldsymbol{2}}}\boldsymbol{V}$ where $\boldsymbol{V}$ is the right singular vector of $\boldsymbol{S}$. The factorial score of the first axis is then defined by $F_{i}^{1}=\frac{1}{K}\sum_{k=1}^{K} \sum_{j_{k}\in\{0,1\}} W_{j_{k}}^{1,k}X_{j_{k},i}^{k}$. The variance explained by the $a^{th}$ factorial axis is given by the eigenvalues $\lambda_{a}\boldsymbol{=}{\boldsymbol{(D}_{s}^{\boldsymbol{2}}\boldsymbol{)}}_{\boldsymbol{a}}$**.**

**1.3 Construction of the composite readiness score**

Following the approach proposed by Asselin (2009), for each indicator $k$ we define a discrimination measure $\Delta_{l}^{a}$ on each factorial axis $a$, $\Delta_{k}^{a}$= $\sum_{j_{k}\in\left\{ 0,1 \right\}} \frac{n_{j_{k}}^{k}}{N}{{(W}_{j_{k}}^{a,k})}^{2}$ where $n_{j_{k}}^{k}$ is the absolute frequency of the $j_{k}$th category of indicator $k$ [3] The average of the discrimination measures across the $K$ indicators on the $a^{th}$ axis corresponds to the total variance explained by the axis, that is, $\lambda_{a}\boldsymbol{=}\frac{1}{K} \sum_{k=1}^{K} \Delta_{k}^{a}$.

For each factorial axis, we split the indicators in two groups, each satisfying the Global First Axis Ordering Consistency condition (FAOC-G) in one of the two axis orientations, i.e. positive $(G_{1})$ or negative $(G_{2})$. We then calculate the total variance explained by each group in the axis, that is, $\Delta_{G_{j}}^{a}= \sum_{k\in G_{j}} \Delta_{k}^{a}$ where $j=1,2$ and retain on the axis the group of indicators explaining more variation that a threshold $T_{a}$ which is taken to be 50% of the variance explained by the axis, that is, $T_{a}=0.5*K*\lambda_{a}$. The groups of indicators retained on the axes, are overlapping and an indicator can be retained on several axes. We remove intersections by selecting the factorial axis with the highest discrimination measure for than indicator among all axes. We define the composite readiness score $F_{i}=\frac{1}{K}\sum_{k=1}^{K} \sum_{j_{k}\in\{0,1\}} \sum_{a=1}^{L} \delta(k-a)W_{j_{k}}^{a,k}X_{j_{k},i}^{k}$ where $L$ is the number of factorial axes used in the composite score and $\delta(k-a)$ is the Dirac delta function which takes the value 1 when the $k^{th}$ indicator is retained on the $a^{th}$ factorial axis and 0 otherwise, that is,$\delta\left( k-a \right)=1$ if $k=a$ and $\delta\left( k-a \right)=0$ if $k\neq a$. To improve interpretation of the score we translate the weights so that the absence category $\left( j_{k}=0 \right)$ of the $k$ indicator to receive a zero weight and the presence one $\left( j_{k}=1 \right)$ to receive a strictly positive representing the gain in the readiness increase measured by the axis $a$ when a facility $i$ acquires the $k$ tracer. Therefore the $W_{j_{k}}^{a,k}$ in $F_{i}$ is replaced by $W_{j_{k}}^{+a,k}$ where $W_{0}^{+a,k}=0$ and $W_{1}^{+a,k}$=$W_{1}^{+a,k}-W_{0}^{a,k}$.

**1.4: Geostatistical variable selection**

To identify the most important readiness tracer items related to malaria deaths, Bayesian geostatistical variable selection was implemented using stochastic search and adopting a spike and slab prior distributions for the regression coefficients [4]. For every readiness indicator $I_{k}$ a Bernoulli variable $\gamma_{k}$ was introduced with Bernoulli probability $\pi_{k}$ corresponding to the inclusion of $I_{k}$ in the model. For the coefficient $\beta_{k}$, we assume a prior distribution which is mixture of non-informative normal distributions, $\beta_{k}\sim\delta\left( \gamma_{k-1} \right)N\left( 0,\tau_{k}^{2} \right)+\left( 1-\delta\left( \gamma_{k-1} \right) \right)N\left( 0,\vartheta_{0}\tau_{k}^{2} \right)$ where $\delta\left( . \right)$ is the Dirac delta function. Therefore, in case $I_{k}$ is included in the model (slab) and an informative normal prior shrinking $\beta_{k}$ to zero (spike) if $I_{k}$ is included in the model, $\beta_{k}\sim N\left( 0,\tau_{k}^{2} \right)$ (slab) and in case $I_{k}$ is excluded, $\beta_{k}\sim N\left( 0,\vartheta_{0}\tau_{k}^{2} \right)$ where $\vartheta_{0}={10}^{5}$ is a very large number shrinking the variance to zero i.e. spike component of the prior. We have adopted a $Beta(1,1)$hyperprior for $\pi_{k}$ and an inverse gamma prior for the variance $\tau_{k}^{2}$ with mean 1 and variance 10.

**References**

1. Cressie. Statistics for Spatial Data, Revised Edition [Internet]. 2015 [cited 2016 Dec 9]. Available from: <http://www.wiley.com/WileyCDA/WileyTitle/productCd-1119114616.html>
2. Gelman A, Rubin DB. Inference from Iterative Simulation Using Multiple Sequences. Stat Sci. 1992;7:457–72.
3. Asselin L-M. Analysis of multidimensional poverty: Theory and case studies. Vol. 7. Springer Science & Business Media; 2009.
4. Chammartin F, Hürlimann E, Raso G, N’Goran EK, Utzinger J, Vounatsou P. Statistical methodological issues in mapping historical schistosomiasis survey data. Acta Trop. 2013 Nov;128:345–52.
