# Supplementary material for: Constructing a malaria-related health service readiness index and assessing its association with child malaria mortality: an analysis of the Burkina Faso 2014 SARA data
Source: BMC Public Health. 2021 Jan 5;21:20. doi: 10.1186/s12889-020-09994-7 (PMC7784320; doi:10.1186/s12889-020-09994-7)
Supplement: Supplementary file 2 — Additional file 2. Supplementary Tables [file 12889_2020_9994_MOESM2_ESM.docx]

Table 2.1: Selection of factorial axes included in the composite score for medical centers.

| **Tracers** | **Discrimination measures** | | | | | | | | | | | | | | **Selected factorial axes** | Weights^b^  $\mathsf{W}_{1}^{+\alpha, k}$ |
| --- | --- | --- | --- | --- | --- | --- | --- | --- | --- | --- | --- | --- | --- | --- | --- | --- |
|  | Factorial axes^a^ | | | | | | | | | | | | | |  |  |
|  | 1 | 2 | 3 | 4 | 5 | 6 | 7 | 8 | 9 | 10 | 11 | 12 | 13 | 14 |  |  |
| Privacy room | **0.004** | **0.049** | 0.003 | **0.150** | 0.036 | **0.038** | **0.001** | 0.009 | 0.000 | **0.387** | **0.128** | 0.032 | 0.001 | 0.017 | 10 | 7280 |
| Emergency transportation | 0.000 | **0.056** | **0.028** | 0.061 | 0.000 | **0.188** | 0.062 | **0.315** | **0.000** | **0.007** | 0.006 | 0.022 | **0.012** | 0.011 | 8 | 11826 |
| Light power | **0.027** | **0.217** | 0.011 | 0.009 | 0.001 | 0.068 | **0.168** | **0.030** | **0.004** | **0.090** | 0.039 | **0.152** | **0.000** | **0.000** | 2 | 4078 |
| Safe final disposal of sharps | 0.072 | **0.012** | **0.502** | **0.171** | **0.002** | 0.007 | **0.003** | **0.014** | 0.017 | **0.004** | 0.048 | **0.002** | **0.008** | **0.000** | 3 | 6399 |
| Safe final disposal of infectious wastes | 0.039 | **0.015** | **0.602** | **0.166** | 0.000 | 0.001 | **0.034** | **0.006** | 0.019 | 0.008 | **0.000** | 0.000 | **0.000** | **0.002** | 3 | 6771 |
| Appropriate storage of infectious waste | 0.018 | **0.054** | 0.000 | 0.006 | **0.015** | **0.377** | **0.006** | **0.001** | **0.007** | 0.014 | 0.099 | **0.106** | 0.039 | 0.026 | 6 | 9848 |
| Latex gloves | **0.001** | **0.119** | 0.006 | 0.007 | **0.118** | **0.005** | **0.259** | 0.077 | **0.057** | 0.001 | 0.024 | 0.002 | **0.045** | **0.008** | 7 | 7596 |
| Guidelines for standard precautions | **0.269** | 0.012 | **0.036** | **0.020** | **0.029** | **0.023** | 0.111 | 0.007 | **0.024** | **0.080** | **0.004** | **0.054** | **0.010** | 0.006 | 1 | 4126 |
| Haemoglobin test | **0.095** | 0.096 | 0.012 | 0.013 | **0.158** | 0.003 | 0.008 | **0.002** | **0.159** | 0.025 | **0.014** | 0.063 | **0.176** | 0.004 | 13 | 5035 |
| Glucose dipstick | **0.090** | 0.002 | **0.098** | 0.000 | **0.185** | 0.027 | 0.047 | 0.068 | **0.003** | 0.088 | 0.000 | **0.067** | **0.000** | 0.000 | 5 | 6889 |
| Amlopdipin | **0.054** | **0.162** | 0.029 | **0.030** | **0.016** | **0.032** | **0.117** | 0.002 | 0.169 | 0.015 | **0.026** | 0.000 | 0.019 | 0.003 | 2 | 3238 |
| Aspirin | **0.336** | 0.163 | **0.000** | **0.005** | 0.018 | **0.015** | 0.006 | 0.038 | **0.051** | 0.015 | 0.000 | **0.048** | 0.023 | 0.016 | 1 | 4151 |
| Beclomethasone inhaler | **0.043** | **0.290** | 0.021 | **0.213** | **0.002** | **0.000** | 0.028 | **0.030** | **0.036** | 0.025 | 0.058 | 0.000 | 0.025 | **0.001** | 2 | 5464 |
| Betablockers | **0.125** | **0.257** | 0.116 | **0.063** | **0.009** | 0.021 | 0.068 | **0.004** | 0.003 | **0.001** | 0.002 | 0.000 | 0.027 | 0.047 | 2 | 4441 |
| Ceftriaxone | **0.517** | 0.009 | 0.006 | **0.004** | 0.053 | **0.016** | **0.022** | **0.033** | 0.019 | **0.002** | 0.041 | 0.003 | **0.114** | 0.009 | 1 | 6950 |
| Gentamicin | **0.296** | **0.016** | **0.036** | 0.133 | 0.052 | 0.029 | **0.002** | **0.003** | 0.015 | 0.013 | **0.004** | **0.019** | **0.000** | 0.135 | 1 | 4803 |
| Glibenclamide | **0.142** | **0.007** | **0.042** | 0.042 | 0.047 | 0.000 | **0.003** | **0.008** | **0.141** | **0.042** | 0.008 | 0.019 | 0.193 | **0.203** | 14 | 5520 |
| Insulin injectable | **0.028** | **0.208** | 0.043 | **0.027** | **0.009** | **0.015** | 0.148 | 0.001 | 0.073 | 0.026 | **0.053** | **0.059** | **0.017** | **0.157** | 2 | 5936 |
| Magnesium | **0.274** | **0.159** | **0.003** | 0.054 | 0.005 | 0.008 | **0.079** | 0.000 | 0.002 | **0.002** | **0.084** | 0.012 | 0.001 | **0.013** | 1 | 2990 |
| Oxytocin | **0.197** | **0.011** | **0.109** | 0.227 | **0.005** | 0.060 | 0.011 | **0.036** | 0.050 | 0.006 | **0.019** | **0.043** | 0.004 | **0.000** | 1 | 3530 |
| Salbutamol | **0.394** | **0.007** | **0.024** | **0.033** | **0.015** | **0.007** | **0.001** | 0.029 | **0.050** | 0.038 | **0.001** | 0.031 | 0.007 | 0.009 | 1 | 3908 |
| zinc | **0.306** | 0.010 | **0.007** | **0.026** | 0.000 | 0.123 | 0.005 | **0.013** | 0.024 | 0.006 | 0.040 | 0.120 | 0.030 | 0.010 | 1 | 3137 |
| ITN | **0.020** | 0.001 | **0.107** | **0.011** | 0.098 | **0.176** | **0.025** | **0.000** | **0.031** | 0.066 | **0.181** | **0.014** | 0.002 | **0.001** | 11 | 5142 |
| Staff trained in malaria diagnosis and treatment | **0.072** | 0.096 | **0.003** | **0.001** | **0.302** | **0.012** | **0.026** | 0.069 | 0.081 | **0.000** | 0.001 | 0.001 | 0.023 | **0.000** | 5 | 4971 |
| Staff trained in IPTg | **0.002** | **0.010** | **0.024** | 0.207 | **0.061** | **0.220** | 0.009 | 0.012 | 0.063 | **0.042** | 0.021 | 0.102 | 0.013 | 0.000 | 6 | 4323 |
| First line treatment of malaria | **0.403** | 0.099 | 0.005 | **0.015** | 0.000 | **0.014** | 0.004 | 0.002 | 0.029 | 0.017 | 0.029 | 0.001 | **0.003** | **0.088** | 1 | 5211 |
| IPTg drug | **0.348** | 0.095 | 0.017 | **0.028** | 0.128 | **0.033** | **0.032** | 0.000 | 0.006 | **0.020** | 0.034 | **0.001** | **0.056** | 0.010 | 1 | 6019 |
| Carbamazepine | **0.009** | 0.156 | 0.027 | **0.037** | **0.194** | **0.002** | **0.050** | 0.175 | 0.016 | **0.002** | 0.050 | **0.000** | **0.029** | 0.036 | 5 | 4198 |
| Haloperidol | **0.015** | 0.191 | 0.023 | **0.008** | **0.148** | **0.005** | **0.025** | **0.304** | **0.000** | **0.015** | **0.018** | **0.013** | **0.001** | 0.040 | 8 | 6119 |
| Variance Threshold ($T_{a}$) | 2.099 | 1.289 | 0.971 | 0.883 | 0.853 | 0.762 | 0.680 | 0.644 | 0.574 | 0.529 | 0.516 | 0.492 | 0.438 | 0.427 |  |  |
| Variation explained ($\Delta_{G1}^{a}$) | **4.068** | **1.746** | **1.634** | **1.007** | **1.269** | **1.174** | **0.974** | **0.610** | **0.578** | **0.780** | **0.583** | **0.577** | **0.442** | **0.519** |  |  |
| Variation explained ($\Delta_{G2}^{a}$) | 0.130 | 0.831 | 0.308 | 0.759 | 0.437 | 0.350 | 0.315 | 0.315 | 0.571 | 0.278 | 0.449 | 0.407 | 0.435 | 0.334 |  |  |
| Variation explained after eliminating intersection axes | 3.340 | 1.133 | 1.104 | 0.000 | 0.682 | 0.597 | 0.259 | 0.620 | 0.000 | 0.387 | 0.181 | 0.000 | 0.176 | 0.203 |  |  |

^a^Not bold cells: Group 1, negative orientation; Bold: Group 2, positive orientation

^b^Weights were multiplied by 1000

Table 2.2: Selection of factorial axes included in the composite score for peripheral health centers

| Tracers | Discriminant measures^a^ | | | | | | **Selected factorial axes** | Weights^b^  $\mathsf{W}_{1}^{+\alpha, k}$ |
| --- | --- | --- | --- | --- | --- | --- | --- | --- |
|  | Factorial axes | | | | | |  |  |
|  | 1 | 2 | 3 | 4 | 5 | 6 |  |  |
| Improved water source | 0.006 | 0.000 | **0.455** | 0.001 | **0.471** | 0.000 | 5 | 6537 |
| Emergency transportation | **0.400** | **0.003** | **0.010** | **0.004** | **0.013** | 0.066 | 1 | 6117 |
| Soap or running water | **0.017** | **0.006** | 0.411 | **0.186** | **0.318** | **0.002** | 5 | 8143 |
| Storage infectious waste | 0.008 | **0.651** | **0.004** | 0.033 | **0.003** | 0.001 | 2 | 7308 |
| Latex gloves | 0.003 | **0.656** | 0.018 | 0.031 | **0.000** | 0.000 | 2 | 7677 |
| Urine dipstick | **0.448** | 0.004 | **0.005** | 0.024 | **0.009** | 0.034 | 1 | 5448 |
| Ceftriaxone | **0.312** | **0.027** | **0.026** | **0.028** | 0.141 | 0.026 | 1 | 4186 |
| Oxytocin | **0.579** | **0.000** | 0.018 | 0.007 | **0.007** | 0.003 | 1 | 6254 |
| Thiazidic | 0.006 | **0.063** | **0.089** | **0.716** | 0.022 | 0.002 | 4 | 10001 |
| IPTg training | **0.219** | **0.005** | **0.015** | **0.001** | 0.001 | **0.745** | 6 | 6232 |
| Variance Threshold ($T_{a}$) | 0.999 | 0.708 | 0.526 | 0.515 | 0.494 | 0.439 |  |  |
| Variation explained ($\Delta_{G1}^{a}$) | **1.9752** | **1.411** | **0.606** | **0.935** | **0.823** | **0.747** |  |  |
| Variation explained ($\Delta_{G2}^{a}$) | 0.023 | 0.004 | 0.447 | 0.096 | 0.164 | 0.131 |  |  |
| Variation explained after eliminating intersection axes | 1.740 | 1.308 | 0.000 | 0.716 | 0.790 | 0.745 |  |  |

^a^Not bold cells: Group 1, negative orientation; Bold: Group 2, positive orientation

^b^Weights were multiplied by 1000
